# Unknown and Unacknowledged Dangers to Every Medical Student: A Rare Case of Nitric Acid Burns

**DOI:** 10.7759/cureus.52203

**Published:** 2024-01-13

**Authors:** Kavyanjali Reddy, Pankaj Gharde, Harshal Tayade, Mihir Patil, Lucky Srivani Reddy, Dheeraj Surya

**Affiliations:** 1 Surgery, Jawaharlal Nehru Medical College, Datta Meghe Institute of Higher Education and Research, Wardha, IND; 2 Obstetrics and Gynaecology, Jawaharlal Nehru Medical College, Datta Meghe Institute of Higher Education and Research, Wardha, IND

**Keywords:** preventive measures, emergency medicine, occupational hazards, medical students, nitric acid, chemical burns

## Abstract

This case report delves into the often overlooked and unacknowledged hazards faced by medical students, exemplified by a rare incident of nitric acid burns. A 19-year-old male medical student with no notable medical, surgical, or familial history suffered a spillage of 69% nitric acid on the anterior aspect of the right thigh while engaged in laboratory work. Swift action, including immediate wound irrigation, application of silver sulfadiazine, and subsequent hospitalization, proved crucial in mitigating the burn's severity. Though vitally stable, the patient exhibited a distinctive color change in the wound during observation. Admitted to the general surgery ward, outpatient follow-ups revealed successful wound healing within four weeks, emphasizing the importance of prompt intervention and meticulous care in addressing chemical burn injuries among medical students. This report sheds light on the often-underestimated dangers inherent in pursuing medical education.

## Introduction

Chemical burn injuries pose unique challenges in the realm of emergency medicine and require prompt and specialized care. Nitric acid, a potent corrosive substance, can lead to severe tissue damage upon contact, making incidents involving its exposure a matter of considerable concern [1]. Often engrossed in laboratory activities, medical students are susceptible to such occupational hazards, which warrant heightened awareness and preventative measures [2].

In this context, chemical burns merit meticulous evaluation and immediate intervention to prevent complications such as infection, delayed wound healing, or long-term sequelae [3]. The initial management of chemical burns typically involves thorough decontamination and wound care, aiming to minimize the extent of tissue damage [4].

This case report focuses on a rare incident involving a 19-year-old male medical student who sustained nitric acid burns during laboratory work. The objective is to highlight the potential dangers medical students face, emphasizing the importance of preventative measures and timely, effective management in the event of chemical exposures.

## Case presentation

**Figure 1:**
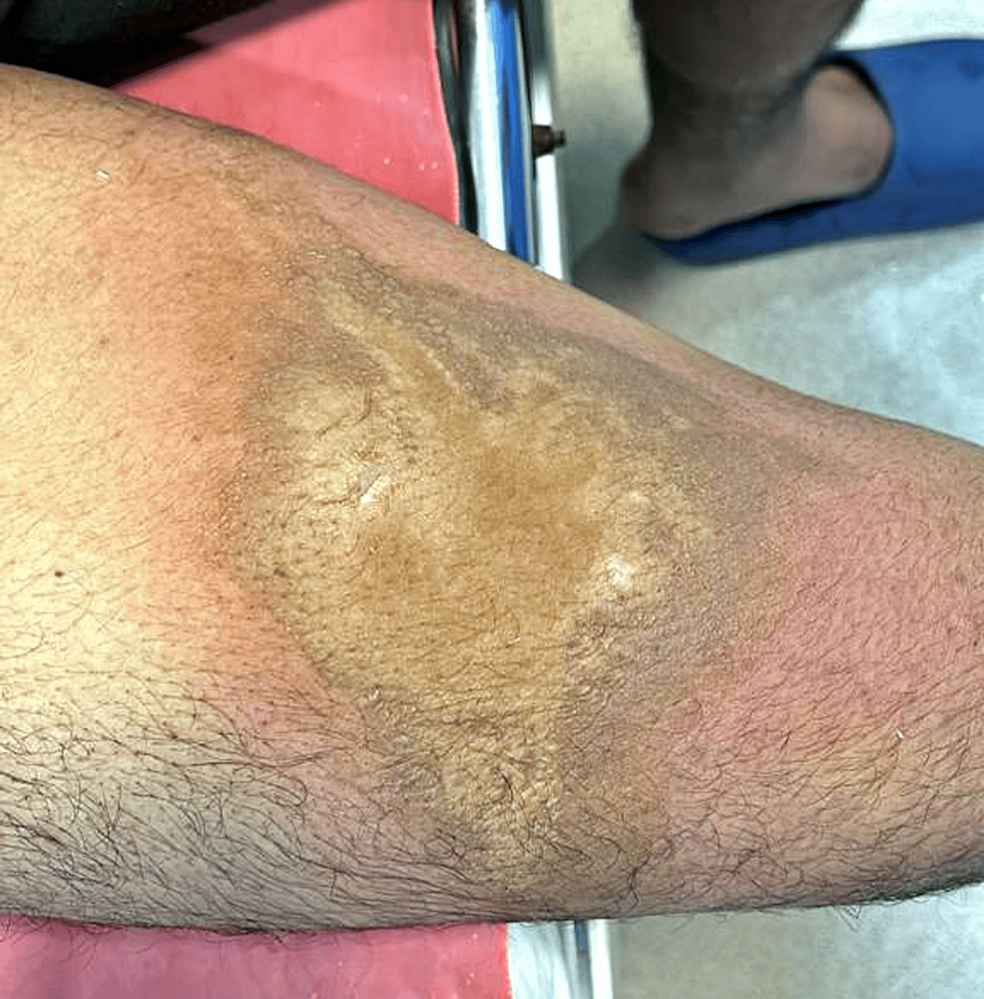
Shows yellowish hue on the burnt area immediately after the incident

A 19-year-old male medical student with no significant medical, surgical, or family history presented to the emergency medicine department, reporting an incident involving the spillage of 69% nitric acid on the anterior aspect of his right thigh while working in a biochemistry lab. He arrived at the emergency department 15 minutes after the accident with a characteristic nitric acid burn wound, classified as between first and second-degree Figure 1.

The patient maintained vital stability, with a pulse rate of 98 beats per minute and a blood pressure of 120/70 mm Hg. His Glasgow Coma Scale (GCS) was E4V5M6. The patient reported washing the wound under tap water for 5 minutes before seeking medical attention. Subsequently, the wound received a thorough saline wash for 15 minutes. A distinctive yellowish hue was observed over the thigh, prompting the application of silver sulfadiazine ointment and coverage with a Bactigras dressing (Smith & Nephew plc, Watford, UK).

**Figure 2:**
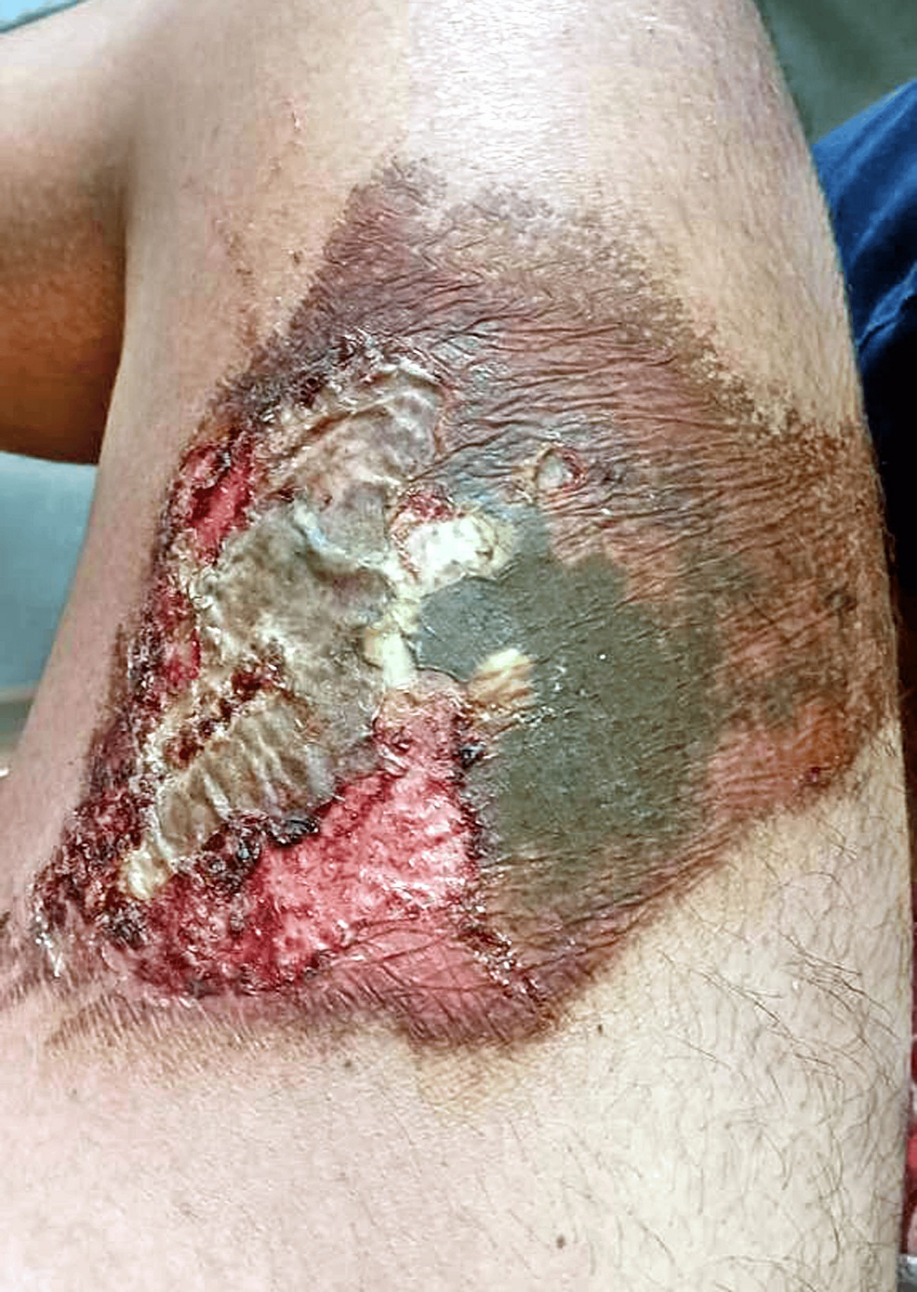
Burn wound with characteristic green colour 24 hours after the incident

An electrolyte panel, routine blood investigations, and an electrocardiogram (ECG) revealed results within normal limits. The patient was admitted to the general surgery ward for observation. After 24 hours, the wound exhibited a peculiar green colour (Figure 2).

**Figure 3:**
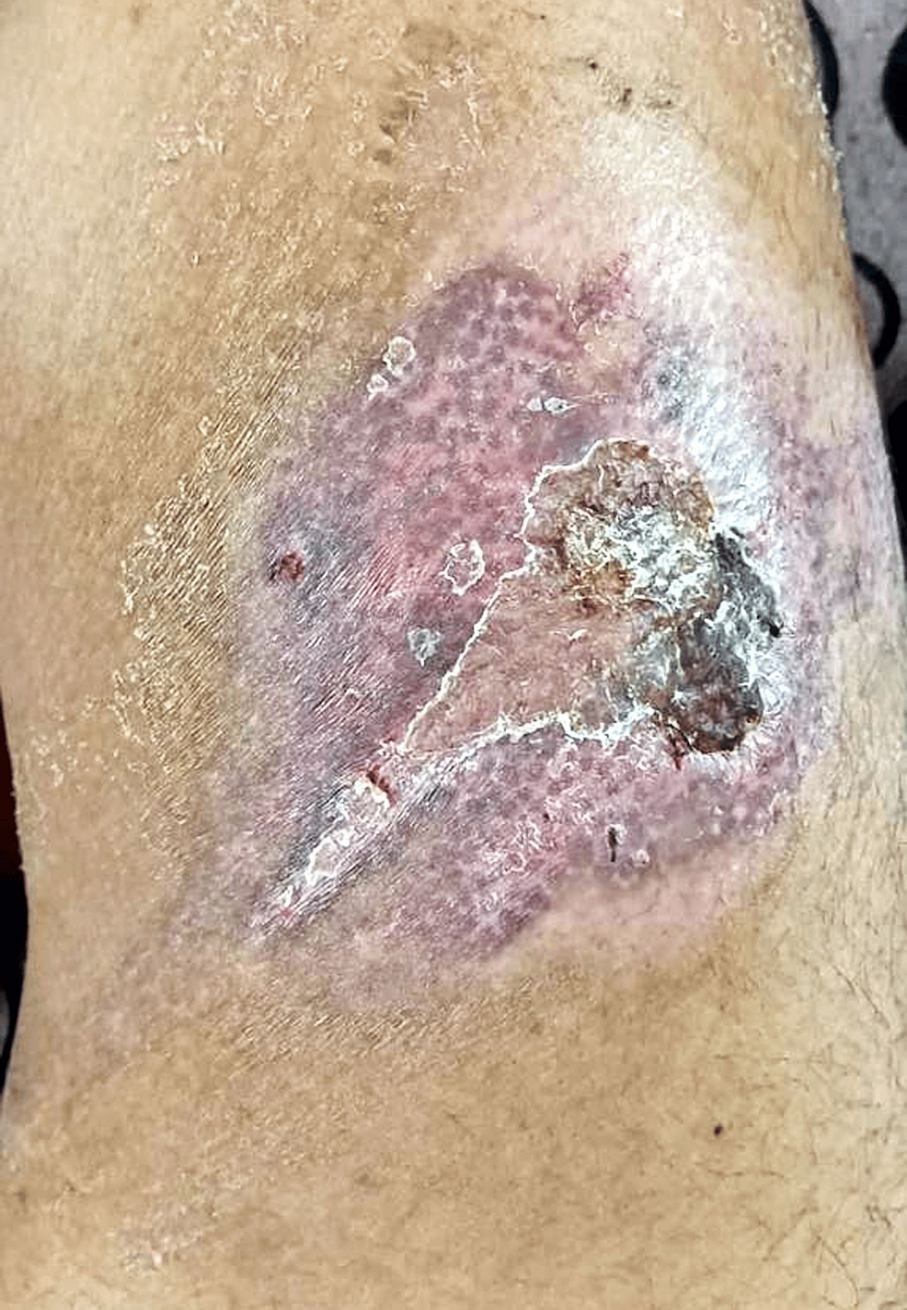
Shows healing with scar formation after four weeks with no signs of infection

Upon discharge, the patient was advised to follow up in the surgery outpatient department for further assessment and dressing. Over four weeks, the wound demonstrated healing with scar formation, devoid of signs of inflammation or infection Figure 3.

## Discussion

The presented case report underscores the significance of prompt and comprehensive management in the context of a nitric acid burn injury. Chemical burns, particularly those involving corrosive substances like nitric acid, necessitate meticulous evaluation and intervention to mitigate potential complications. The patient, a 19-year-old male medical student, experienced a spillage incident in the biochemistry lab, resulting in a nitric acid burn to the anterior aspect of the right thigh. The initial steps of wound irrigation and subsequent application of silver sulfadiazine ointment align with established protocols for managing chemical burns [5]. The importance of immediate decontamination, as evidenced by the patient washing the wound under tap water before seeking medical attention, cannot be overstated [6]. This rapid response likely contributed to the observed vitally stable condition upon arrival at the emergency department.

The peculiar color changes in the wound, evolving from yellowish to green after 24 hours, may be attributed to the nature of the chemical exposure and subsequent healing processes [7]. Similar color changes in chemical burns have been reported in the literature, highlighting the dynamic nature of such injuries [8]. Laboratory investigations, including the electrolyte panel and routine blood tests, were crucial in assessing the patient's overall health status and guiding further management. The normal limits observed in these tests are consistent with the findings of other studies emphasizing the importance of systemic evaluation in burn patients [9].

The decision to admit the patient to the general surgery ward for observation aligns with the cautious approach recommended in cases of chemical burns, ensuring close monitoring for any signs of complications [10]. The subsequent outpatient follow-ups facilitated ongoing assessment, dressing changes, and monitoring of wound healing. The successful outcome of this case, with wound healing and scar formation within four weeks, underscores the effectiveness of the implemented management strategy. The absence of signs of inflammation or infection during the follow-up period further supports the appropriateness of the chosen interventions.

## Conclusions

In conclusion, this case report sheds light on the need for heightened awareness and precautionary measures among medical students who face potential occupational hazards such as chemical burns while immersed in laboratory activities. The timely and comprehensive management of a rare incident involving nitric acid burns in a 19-year-old male medical student underscores the importance of swift intervention, including immediate wound irrigation and specialized care. The observed color changes in the wound and subsequent successful healing within four weeks emphasize the dynamic nature of chemical burns and the positive impact of meticulous treatment. This case highlights the often-underestimated occupational risks medical students face. It underscores the significance of preventive strategies, prompt action, and continuous education to ensure the well-being of those pursuing medical education. Further research and awareness campaigns are warranted to enhance safety protocols and minimize the occurrence of such incidents in educational settings.
